# Public health round-up

**DOI:** 10.2471/BLT.24.010124

**Published:** 2024-01-01

**Climate and health**

People wade through floodwater in the town of Beledweyne in Somalia, one of many countries affected by climate change in 2023. At the 28^th^ United Nations Climate Change Conference, more than 40 million health professionals from around the globe joined the call to action by the World Health Organization (WHO) and civil society organizations, to prioritize health in climate negotiations.

**Figure:**
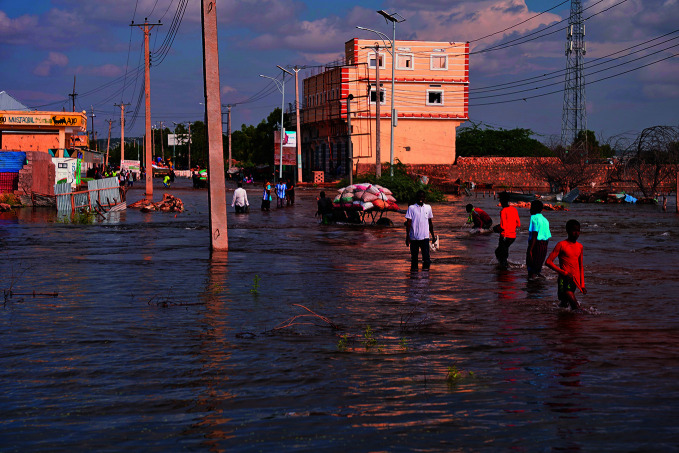
UNICEF/Maslah Mumin

## Health on the climate change agenda

The 28th meeting of the Conference of the Parties (COP 28) to the United Nations (UN) Framework Convention on Climate Change included the first-ever day dedicated to the topic of health. The 3 December event, which took place in Dubai, in the United Arab Emirates, included presentations of the evidence linking climate change and human health, health arguments for climate action, and the health benefits to be derived from climate change mitigation.

World Health Organization (WHO) Director-General Tedros Adhanom Ghebreyesus told delegates that the inclusion of health on the climate agenda was long overdue.

On 23 November, WHO published a report on the progress governments have made in addressing the health risks of climate change. On 2 December, a consortium of multilateral development banks, funders, governments and philanthropies published *Guiding principles for financing climate and health solutions. *Both publications emphasized the need to rapidly reduce greenhouse gas emissions, protect people from the range of climate risks to health, and build resilient, environmentally sustainable health systems.

https://bit.ly/3Tfj3Zy

https://bit.ly/3GzMmhR

https://bit.ly/3TbuHEL

https://bit.ly/3T9e2BI

## Responding to the health emergency in Gaza

A Special Session of the WHO Executive Board was held on 10 December 2023. WHO Member States adopted a resolution calling on the Director-General to report on the public health implications of the crisis in Gaza, and for WHO to strengthen its assistance to the affected population.

In his opening remarks, the Director-General deplored the attacks on Israel of 7 October 2023 and provided an update on the situation in Gaza where, as of 10 December, more than 17 000 people were reported to have died, including 7000 children; while more than 46 000 people were reported to have been injured. He also drew attention to the state of Gaza’s health system, noting that only 14 hospitals of the original 36 are even partially functional.

Some 1.9 million people were reported to have been displaced, and were being concentrated into a small area in the south of Gaza where overcrowding, combined with the lack of adequate food, water, shelter, sanitation and health care were creating conditions where diseases were likely to spread.

The Director-General echoed the call made a week earlier by UN Secretary-General António Guterres for a humanitarian ceasefire, and regretted the failure of the UN Security Council on 8 December to adopt a resolution demanding a ceasefire and the immediate and unconditional release of hostages, as well as assurance of humanitarian access. In his closing remarks, he thanked the Executive Board for passing the resolution, but reiterated that in the current context, sustained humanitarian assistance at the scale needed is simply not possible. “Without a ceasefire, there is no peace. And without peace, there is no health,” he said.

https://bit.ly/47135Wg

https://bit.ly/46NqPNr

## Malaria rising

WHO’s latest estimates of malaria incidence show an increase of 16 million annual cases, despite expanding access to insecticide-treated nets and medicines to help prevent malaria in young children and pregnant women. A report published by WHO on 30 November registers an estimated 249 million malaria cases globally in 2022, an increase from the 2019 estimate of 233 million.

In addition to the disruptions caused by coronavirus disease 2019 (COVID-19), the global malaria response has faced drug and insecticide resistance, humanitarian crises, resource constraints, climate change impacts and delays in programme implementation, particularly in countries with a high burden of the disease.

https://bit.ly/3TaHszn

## Anthrax in Zambia

The International Health Regulations national focal point of Zambia notified WHO of an anthrax outbreak in humans. As of 20 November 2023, 684 suspected human cases, including four deaths (case fatality rate 0.6%) had been reported across nine of Zambia’s 10 provinces.

According to an 8 December WHO disease outbreak news report, efforts to control the outbreak are being driven by several factors, including limited community knowledge regarding anthrax transmission; high levels of poverty and food insecurity; a shortage of available vaccines and laboratory reagents; inadequate carcass disposal; and decontamination practices.

WHO is working closely with the UN Food and Agriculture Organization and the Ministry of Agriculture to respond to the outbreak, and a joint One Health task force has been conducting case-finding in animals and humans.

https://bit.ly/3RkHUIQ

## Acute malnutrition guidance

WHO launched a new guideline on the prevention and management of wasting and nutritional oedema (acute malnutrition) which continues to affect an estimated 45 million children under five worldwide.

Published on 20 November, it focuses on both prevention and management of acute malnutrition, and highlights the vital importance of investing in both these aspects. The guideline provides evidence-based recommendations and will be followed by guidance and tools for implementation.

WHO Director-General Tedros stressed the importance of preventing and managing acute malnutrition as part of a continuum of care, saying, “we are calling for more integration of nutrition services into health systems and the strengthening of those health systems.”

https://bit.ly/3uPWIHJ

## Managing chronic low back pain

WHO released guidelines on managing chronic low back pain in primary and community care settings, listing interventions for health workers to use and also to not use during routine care.

Low back pain is a leading cause of disability globally. In 2020, approximately 1 in 13 people, equating to 619 million people, experienced low back pain, a 60% increase from 1990. Cases are expected to rise, commensurate with population growth, to an estimated 843 million by 2050.

The WHO guidelines focus on non-surgical interventions to help people experiencing chronic primary low back pain, including education for self-care strategies and non-steroidal anti-inflammatory medicines.

https://bit.ly/3TeDwxz

**Cover photo**

A woman at an open-air market in Mutanpal village in Chhattisgarh, India, a state where malaria rates have declined sharply since 2016.

**Figure:**
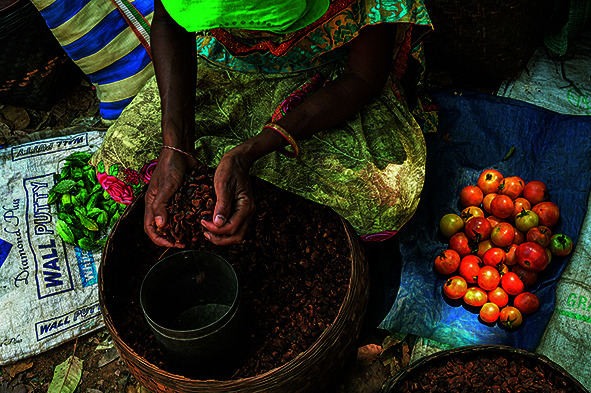
WHO/Atul Loke/Panos Pictures

## Tackling road traffic deaths

WHO and partners launched a revised *Speed management manual* to help avert road traffic deaths and injuries due to speeding.

Launched on 5 December, the manual includes new case studies; data and guidance for decision-makers on setting and enforcing speed limits; modifying roads to reduce speed; raising awareness of the dangers of speeding; making use of in-vehicle technologies; and tracking the impact of policies.

https://bit.ly/4abanZZ

## Taxing alcohol and sugar-sweetened drinks

WHO released new global data showing a low level of taxes being applied to unhealthy products such as alcohol and sugar-sweetened beverages. The data indicate that most countries are not using such taxes to incentivize healthy behaviour. WHO also released a manual on alcohol tax policy and administration.

It is estimated that globally 2.6 million people die from drinking alcohol every year, and over 8 million die from an unhealthy diet. Taxes on alcohol and sugar-sweetened beverages have been shown to change consumption levels of these products.

https://bit.ly/46Nbv3d

## Long term postpartum morbidity

Every year, at least 40 million women are likely to experience a long-term health problem caused by childbirth. This is according to a new study published on 7 December in *The Lancet Global Health*.

The first in a series of four papers on maternal health that was supported by the United Nations Special Programme on Human Reproduction, WHO and the United States Agency for International Development, the study reveals the high prevalence of postnatal conditions that persist in the months or even years after giving birth. The problems include pain during sexual intercourse, affecting more than a third of postpartum women, low back pain, incontinence and depression.

https://bit.ly/4ac9CQp

**Looking ahead**

15–19 January. World Economic Forum Annual Meeting: From Lab to Life: Science in Action. Davos, Switzerland. https://bit.ly/3MXbN0j

22–27 January. The Prince Mahidol Award Conference 2024. Geopolitics, human security and health equity in an era of polycrises. Bangkok, Thailand. https://pmac2024.com/

22–27 January. 154th Session WHO Executive Board meeting. WHO headquarters, Geneva, Switzerland. https://bit.ly/3ThRv5S

